# The endocrine disruptor DEHP and the ECS: analysis of a possible crosstalk

**DOI:** 10.1530/EC-19-0548

**Published:** 2020-01-06

**Authors:** Jana Ernst, Urszula Grabiec, Kathrin Falk, Faramarz Dehghani, Kristina Schaedlich

**Affiliations:** 1 Department of Anatomy and Cell Biology, Faculty of Medicine, Martin Luther University Halle-Wittenberg, Halle (Saale), Germany

**Keywords:** adipocytes, DEHP, endocannabinoid system, leptin, SGBS, endocrine disruptor

## Abstract

Studies of the last decade associated the environmental contamination by di-(2-ethylhexyl)-phthalate (DEHP) with obesity and endocrine malfunction. DEHP was found to interact with several receptors – among them are receptors of the endocannabinoid system (ECS) with high expression levels in adipose tissue. Furthermore, the correlation for BMI and body fat to the serum endocannabinoid level raises the question if the obesogenic and endocrine-disrupting DEHP effects are mediated via the ECS. We therefore characterized the ECS in a human cell model of adipogenesis using the SGBS preadipocytes to subsequently investigate if DEHP exposure affects the intrinsic ECS. The receptors of the ECS and the endocannabinoid-metabolizing enzymes were upregulated during normal adipogenesis, accompanied by an increasing secretion of the adipokines adiponectin and leptin. DEHP affected the secretion of both adipokines but not the ECS, suggesting DEHP to alter the endocrine function of adipocytes without the involvement of the intrinsic ECS.

## Introduction

During the last decades, environmental pollution as well as the prevalence of obesity and metabolic syndrome have increased (1). So, an environmental link to obesity is barely surprising (2, 3, 4, 5). Increasing evidence was found for an adverse impact on human health due to environmental agents, such as phthalates (6). Di-(2-ethylhexyl)-phthalate (DEHP) has belonged to the most abundant phthalates in industry and consumer goods for many years acting as an endocrine-disrupting chemical (7). As DEHP is not covalently bound to PVC, it easily migrates into the environment and by that accumulates in lipophilic products like cosmetics or food and binds to house dust particles (8). Associations to numerous health problems, including obesity, diabetes and the metabolic syndrome, have been observed by epidemiological studies (9, 10, 11, 12, 13). Furthermore, investigations in mice identified DEHP as an obesogen by increasing food intake, body weight, fat mass, serum leptin and decreasing serum adiponectin (14, 15, 16). Accordingly, data from *in vitro* models showed a DEHP-dependent impairment of adipogenesis and adipocyte function (14, 17). Analyses on underlying mechanisms are difficult, because energy metabolism and endocrine homeostasis involve complex regulatory systems – among them is the endocannabinoid system (ECS). Endocannabinoids are endogenous ligands of the G-protein-coupled cannabinoid receptors, imitating several effects of the pharmacological active substance Δ9-tetrahydrocannabinol (THC) from cannabis sativa (18). Endocannabinoids like the N-arachidonoylethanolamine (AEA) and 2-arachidonoylglycerol (2-AG) mediate their effects via CB_1_ and CB_2_ cannabinoid receptors (19, 20, 21), but also via the recently identified GPR55 (22) and non-cannabinoid receptors like the transient receptor potential vanilloid 1 (TRPV1) (23) or the peroxisome proliferator-activated receptors (PPARs) (24). Endocannabinoid levels are regulated by the synthesizing enzymes N-acylphosphatidylethanolamine phospholipase D (NAPE-PLD) and diacylglycerol lipase (DAGL) (25, 26) as well as by the metabolizing fatty acid amide hydrolase (FAAH) and monoacylglycerol lipase (MAGL) (27, 28). The influence of phthalates on ECS in peripheral organs is poorly investigated. However, the effect of di-isononyl phthalate (DiNP) as one of the dominating alternatives to DEHP was studied in fish models demonstrating a deregulation of the intrinsic ECS in the gonads, the liver as well in the hepatic lipid metabolism (29, 30, 31). DiNP exhibited adipogenic activity in murine 3T3-derived adipocytes (32).

Adipose tissue expression of ECS components differs between lean and obese subjects as reflected by altered blood endocannabinoid levels (33, 34, 35, 36, 37). Literature in the field of ECS, adipogenesis and adipose tissue as a metabolic and endocrine organ still draw a blurred picture of possible interactions. As CB_1_ activation promotes adipocyte proliferation and differentiation, it furthermore positively affects insulin-stimulated but not basal glucose uptake in 3T3-derived adipocytes (38, 39, 40). Accordingly, an increase of glucose uptake after activation of CB_1_ was demonstrated in human primary adipocytes – accompanied by calcium influx and translocation of GLUT4. However, adiponectin and leptin were not altered (41). The inhibition of CB_1_R in adipocytes directly reduced the leptin secretion in mice. In line with these results, an *in vitro* study in 3T3-derived adipocytes confirmed increased leptin levels after treatment with different CB_1_R agonists that were inhibited by the employment of a CB_1_R inverse agonist (42). In human adipose tissue, no association was found between *CNR1*-mRNA level and adiponectin expression, its secretion or circulating adiponectin (43). After CB_1_ antagonism in rats, a higher adipose gene expression and serum level of adiponectin was detected. This finding was proposed as a consequence of reduced food intake (44). Contrary to the assumption of an indirect effect, the *in vitro* blockade of CB_1_ led to an upregulation of adiponectin in 3T3-derived adipocytes (38, 39, 45). THC also elevated adiponectin gene expression in this cell line. The authors discussed that the variety of different types and concentrations of CB_1_-manipulating agents may likely be responsible for the miscellaneous effects among studies (46). Comparing different ligands of the ECS in human bone marrow derived adipocytes, an exclusive activation of CB_1_-inhibited adipogenesis paralleled by a reduction of adiponectin. Nevertheless, these effects of CB_1_ activation were diminished when ligands not only bound to CB_1_ but also to the non-CB_1_/CB_2_ receptor PPARgamma, which is a crucial transcription factor of adipogenesis (47).

To date there are no reports on the relationship between DEHP and the ECS in obesity. The known fact of interactions of DEHP with receptors of the ECS (48, 49) raised the question whether obesogenic and endocrine-disrupting DEHP effects in adipocytes are mediated via the ECS. For the present study, we first characterized the intrinsic ECS in a human cell model of adipogenesis using the Simpson-Golabi-Behmel Syndrome (SGBS) preadipocytes followed by investigating the impact of DEHP on the ECS as an endocrine modulator of the adipokine system.

## Materials and methods

### Chemicals

DEHP was dissolved in dimethyl sulfoxide (DMSO), both purchased from Sigma-Aldrich, and stored as a 1000-fold stock solution until further use.

### Cell culture

The SGBS preadipocytes were kindly provided by Prof M Wabitsch (Division of Pediatric Endocrinology and Diabetes, Department of Pediatrics and Adolescent Medicine, Ulm University Medical Center, Ulm, Germany). These preadipocytes are a non-immortalized cell model for adipogenesis cultured and differentiated as described previously without modifying the protocols (50, 51). During the induction phase (day 0 to day 4), cells were exposed to a final DEHP concentration of 128 µM (50 µg/mL) and a concentration of 0.1% DMSO in the culture media, whereas controls were run as vehicle controls with 0.1% DMSO only. These experimental conditions are based on *in-vitro* investigations of our group previously performed in murine C3H10-T1/2 mesenchymal stem cells identifying the induction phase as a particularly vulnerable exposure window of adipogenesis (17, 52) and in the SGBS cell model revealing effective but non-toxic concentrations of used substances (53). Furthermore, the herein applied DEHP concentration is considered as environmentally relevant (54, 55, 56). At day 8 of differentiation, all experiments have been finalized. Samples were taken at day 0, 4 and 8 of adipogenesis.

### Quantitative real-time PCR

Quantitative realtime PCR (qRT-PCR) was performed to measure the mRNA expression levels in a StepOnePlus™ Real-Time PCR System (Applied Biosystems). Plasmid standards were generated based on a gene-specific target sequence. Absolute mRNA copies were calculated by quantitative standard curves using serial dilutions (10^6^, 10^5^, 10^4^ and 10^3^) of gene-specific plasmid standards. Assays were run with duplicates of each cDNA sample as well as a no template control (NTC) in a 96-well format for the following genes: *ADIPOR1* and *ADIPOR2*, *CNR1* and *CNR2*, *DAGLalpha*, *FAAH*, *GLUT1* and *GLUT4*, *GPR55*, *LEPR*, *MAGL*, *NAPE-PLD* and *TRPV1*. For normalization, we analyzed the expression of the housekeeping gene TATA-box-binding protein (*TBP*). Absolute mRNA expression was calculated as copy number per 10^3^ molecules *TBP*. The primers and amplicons were as shown in Table 1.

**Table 1:** Primers for quantitative RT-PCR.

Gene	Accession number	Forward primer	Reverse primer	T_m_ (°C)	Amplicon (bp)
*ADIPOR1*	NM_001290629	TGCGGCGGGGAGTTTAGAAG	CGTGTCAGCTTCCCTGTTACT	63	245
*ADIPOR2*	NM_024551	GAGACACGCGGATCAACTCA	GTTGGTGCCCTTTTCTGAGC	60	175
*CNR1*	NM_033181	CTCAGTCATTTTGAGCTCAGCC	GCCATGTCACCTTTGATGTCTTC	60	153
*CNR2*	NM_001841	GCTCCTCATCTGTTGGTTCC	TGACCATGGAGTTGATGAGGC	60	125
*DAGLa*	NM_006133	AGAATGTCACCCTCGGAATG	GGTTGTAGGTCCGCAGGTTA	60	150
*FAAH*	NM_001441	TCAAGGAGTGCTTCACCTACAAG	GTCATAGCTGAACATGGACTGTG	60	164
*GLUT1*	NM_006516	TGGCATCAACGCTGTCTTCT	CTAGCGCGATGGTCATGAGT	60	212
*GLUT4*	NM_001042	ACTGGCCATTGTTATCGGCA	GTCAGGCGCTTCAGACTCTT	60	213
*GPR55*	NM_005683	GGTGCTCTCCCTCCCATT	GCTCACCAGTAGCGGGTAAC	60	172
*LEPR*	NM_002303	ACACCAGAGTGATGCAGGTTT	ATGCTCAAACGTTTCTGGCTTC	62	187
*MAGL*	NM_007283	ATCACCATTCCCCAAATTGA	GATGTACCAGCCCTTCTGGA	60	204
*NAPE-PLD*	NM_198990	TCACGGATCCCATCTTTAGC	TCTCACAGCCACATTTTTGC	60	243
*TBP*	NM_003194	TGTGCTCACCCACCAACAAT	AGTCGTCTTCCTGAATCCCT	60	199
*TRPV1*	NM_080704	TGACCCTCCTGGTGGAGA	CTGCAGCAGGAACTTCACG	60	158

adiponectin receptor 1 and 2 (*ADIPOR1* and *ADIPOR2*), cannabinoid receptor 1 and 2 (*CNR1* and *CNR2*), diacylglycerol lipase alpha (*DAGLa*), fatty acid amide hydrolase (*FAAH*), glucose transporter 1 and 4 (*GLUT1* and *GLUT4*), G protein-coupled receptor 55 (*GPR55*), leptin receptor (*LEPR*), monoacylglycerol lipase (*MAGL*), N-acylphosphatidylethanolamine phospholipase D (*NAPE-PLD*), TATA-box binding protein (*TBP*) and transient receptor potential vanilloid 1 (*TRPV1*).

### Hormone assay

Cell supernatants were collected to measure the concentrations of leptin (high sensitive Leptin ELISA, IBL, Hamburg, Germany) and adiponectin (Quantikine® ELISA Human Total Adiponectin/Acrp30, BioVendor, Kassel, Germany) by ELISA according to manufacturer’s manual. ELISA data were normalized to the protein concentration of individual samples. Protein was isolated using radioimmunoprecipitation assay (RIPA) buffer including protease and phosphatase inhibitors (Roche). Protein concentration was determined by the BioRad Protein Assay (BioRad).

### Western blot

For protein analyses of ECS components, cells were harvested in lysis buffer containing 80 mM Tris, 70 mM sodium dodecyl sulfate (SDS), 0.3 M saccharose, 3 mM sodium orthovanadate and 0.5 mM phenylmethylsulfonyl floride (PMSF) at pH 7.4. Samples of 40 µg protein were separated by a 12.5% (w/v) SDS-polyacrylamid gel before blotting onto nitrocellulose membrane (Protran BA 85, GE Healthcare). Non-specific protein-binding sites were blocked for 30 min with 5% (w/v) milk (Carl Roth, Karlsruhe, Germany) or 10% (v/v) Roti-block solution (Carl Roth) in TBST. For protein detection, primary antibodies against CB_1_, DAGLalpha and DAGLbeta, FAAH, MAGL and NAPE-PLD as well as against beta-ACTIN and GAPDH as housekeeping proteins were used (Table 2). Membranes were incubated for 16 h at 4°C. They were subsequently washed and the horseradish peroxidase-conjugated secondary antibodies (Table 2) were applied for 1 h at room temperature. Chemiluminescence detection was performed by Luminata Forte (Millipore). ImageJ analysis software version 1.46r (National Institutes of Health, Laboratory for Optical and Computational Instrumentation, University of Wisconsin, Madison, WI, USA) was used for the analysis of the intensity of the immunoreactive bands.

**Table 2:** Antibodies for Western blot (WB) and immunohistochemistry (IHC).

	Dilution WB	Dilution IHC	Manufacturer
Primary antibody			
Rabbit polyclonal antibody against CB_1_	0.5 µg/mL	1.65 µg/mL	Cayman, Mississippi, USA
Rabbit pig antibody against DAGLalpha	1:2000	1:200	Frontier Institute, Hokkaido, Japan
Rabbit antibody against DAGLbeta	1:1000	1:100	Thermo Scientific, Dreieich, Germany
Rabbit antibody against human FAAH	1:1000	1:200	Cayman
Rabbit polyclonal antibody against human MAGL	1:1000	1:200	Cayman
Rabbit polyclonal antibody against human NAPE-PLD	1:1000	1:200	Cayman
Mouse antibody against human beta-ACTIN	1:5000		Cell Signaling, Boston, USA
Rabbit antibody against human GAPDH^a^	1:1000		Cell Signaling
Secondary antibody			
Anti-rabbit-IgG	1:20,000		Vektor laboratories, Burlingame, CA
Anti-mouse-IgG	1:10,000		Vektor laboratories
Anti-rabbit-IgG		1:2	DAKO, Hamburg, Germany

^a^This primary antibody is already horseradish peroxidase-conjugated and does not require any secondary antibody.

cannabinoid receptor 1 (CB_1_), diacylglycerol lipase alpha and beta (DAGLalpha and DAGLbeta), fatty acid amide hydrolase (FAAH), glyceraldehyde 3-phosphate dehydrogenase (GAPDH), monoacylglycerol lipase (MAGL), N-acylphosphatidylethanolamine phospholipase D (NAPE-PLD).

### Immunohistochemical staining

Fifty thousand SGBS cells were plated on PLL (Millipore) covered glass plates and treated according to the protocol (see ‘Cell culture’ section). On day 0, 4 and 8 cells were fixed with 4% paraformaldehyde for 25 min. Before staining, the cells were washed with 0.02 M PBS and incubated with goat serum (Sigma-Aldrich) for 30 min. Primary antibodies against CB_1_, DAGLalpha and DAGLbeta, FAAH, MAGL and NAPE-PLD (Table 2) were diluted in 0.05% BSA (Sigma-Aldrich) and incubated overnight. The next day, cells were washed three times with PBS/Triton and incubated with a HRP-labelled secondary antibody diluted in PBS for 1 h. After washing with PBS, cells were covered with 0.05 M Tris buffer and exposed to DAB (Sigma-Aldrich) for 5 min. Finally, hematoxylin (Carl Roth) staining was performed and the plates were covered with Entallan (Millipore).

### Statistical analyses

At least four independent experiments (N) were performed for each group. Data was presented as mean ± s.e.m. For expression analyses during adipogenesis without DEHP exposure, the values of the DMSO control group at day 0 was defined as reference and the ANOVA with the Bonferroni’s *post hoc* test performed. To evaluate differences between the DMSO control group and the DEHP exposure group, an unpaired Student’s *t*-test or the Wilcoxon rank-sum test were used. Data differences were considered as statistically significant at *P* value ≤0.05.

## Results

### DEHP alters the secretion of adiponectin and leptin during adipogenesis

**Figure 1:**
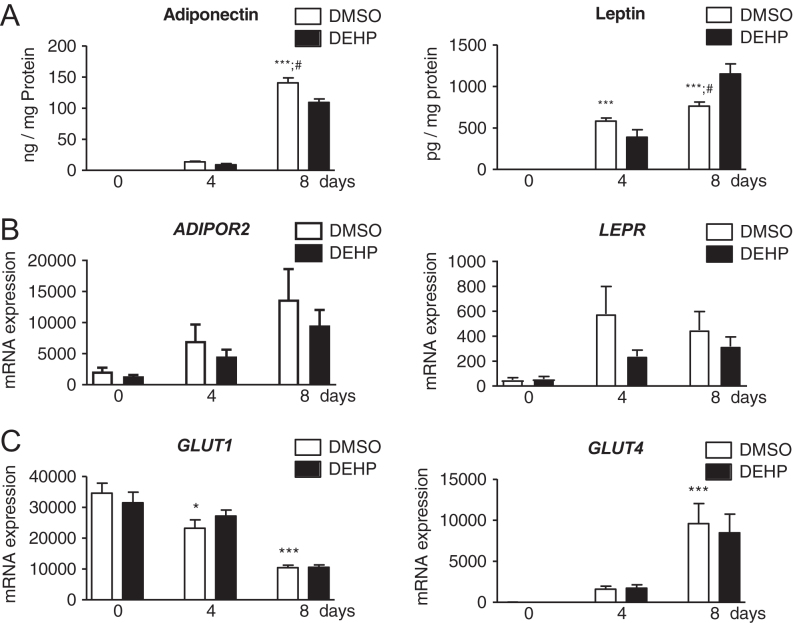
Adipokines, their receptors and the glucose transporters in the SGBS cell model during normal and DEHP-exposed adipogenesis. The secretion of adiponectin and leptin was measured (A). Additionally, the gene expression of their receptors, adiponectin receptor 2 (*ADIPOR2*) and leptin receptor (*LepR*) (B), and the glucose transporters *GLUT1* and *GLUT4* (C) was evaluated in SGBS cells during adipogenic differentiation with and without DEHP. Absolute mRNA expression is presented copy number per 1000 molecules TBP. *n* = 6 for secretion; *n* = 8 for mRNA expression; *P ≤ 0.05; **P ≤ 0.01; ***P ≤ 0.001 for comparing normal adipogenesis to day 0; ^#^*P* ≤ 0.05 for comparing the unexposed and DEHP-exposed group.

To evaluate the efficacy of our experimental conditions on adiponectin and leptin, their receptors and the glucose transporters were investigated as adipogenic markers (Fig. 1). During normal adipogenesis, the secretion level of both adipokines were significantly increased at day 4 and 8 for leptin and at day 8 for adiponectin compared to day 0. DEHP exposure led to significantly reduced adiponectin and increased leptin values at day 8 (Fig. 1A). Gene expression of receptors *ADIPOR2* and *LEPR* was not significantly altered (both with *P* = 0.06 during normal adipogenesis). DEHP had no influence on their gene expression (Fig. 1B). *ADIPOR1* was not expressed. During normal adipogenesis, both investigated glucose transporters showed alterations with a significant decrease of *GLUT1* at day 4 and 8, whereas *GLUT4* increased at day 8. No effect of DEHP exposure was detected for both glucose transporters (Fig. 1C).

### The receptors and the metabolizing enzymes of the ECS were differentially expressed during normal adipogenesis

**Figure 2:**
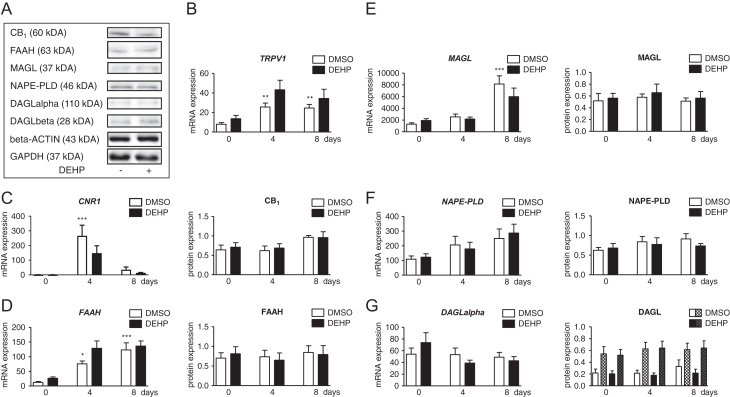
The endocannabinoid system (ECS) in the SGBS cell model during normal and DEHP-exposed adipogenesis. Western blot analyses of CB_1_, FAAH, MAGL, NAPE-PLD, DAGLalpha and DAGLbeta comparing unexposed and mature (day 8) DEHP-exposed SGBS cells were normalized to the endogenous reference beta-ACTIN and GAPDH, respectively (A). Gene and protein expression of the receptors transient receptor potential vanilloid 1 (*TRPV1*) (B) and the cannabinoid receptor 1 (*CNR1*, CB_1_) (C), the enzymes fatty acid amide hydrolase (FAAH) (D), monoacylglycerol lipase (MAGL) (E), N-acylphosphatidylethanolamine phospholipase D (NAPE-PLD) (F) and diacylglycerol lipase alpha (DAGL) (G) were determined in SGBS cells with and without DEHP exposure. For DAGL, the protein expression of both isoforms was evaluated with DAGLalpha as blank columns and DAGLbeta as patterned columns within one figure (G). Absolute mRNA expression is presented copy number per 1000 molecules TBP. *n* ≥ 8 for gene expression; *n* ≥ 4 for protein expression; *P ≤ 0.05; **P ≤ 0.01; ***P ≤ 0.001 for comparing normal adipogenesis to day 0; ^#^*P* ≤ 0.05 for comparing the unexposed and DEHP-exposed group.

The expression of components of ECS was studied during adipogenesis in SGBS cells (Fig. 2). *CNR2* and *GPR55* were absent at detectable expression levels. The expression of the receptor *TRPV1* was significantly upregulated at day 4 and remained at a high level until day 8 (Fig. 2B). Another receptor, *CNR1*, was significantly and transiently upregulated at day 4 and showed a decline at day 8 when compared to day 0 values (Fig. 2C). *FAAH* expression was increased at day 4 and day 8 (Fig. 2D). An elevation in *MAGL* expression was found at day 8 only (Fig. 2E). Data on endocannabinoid-synthesizing enzymes showed no alteration for *NAPE-PLD* and *DAGLalpha* expression (Fig. 2F and G).

### DEHP did not affect the expression of the ECS

After investigating the expression of the ECS during normal adipogenesis, SGBS cells were exposed to DEHP within the induction phase, and its influence on mRNA and protein levels was measured (Fig. 2). DEHP did not significantly change the expression of *TRPV1*, *CNR1*, *FAAH*, *MAGL*, *NAPE-PLD* and *DAGLalpha* (Fig. 2B, C, D, E, F and G). This was verified for CB_1_, FAAH, MAGL, NAPE-PLD and DAGL at protein level (Fig. 2C, D, E, F and G). Western blots for CB_2_, GPR55 and TRPV1 could not be performed due to a lack of appropriate specific antibodies.

**Figure 3:**
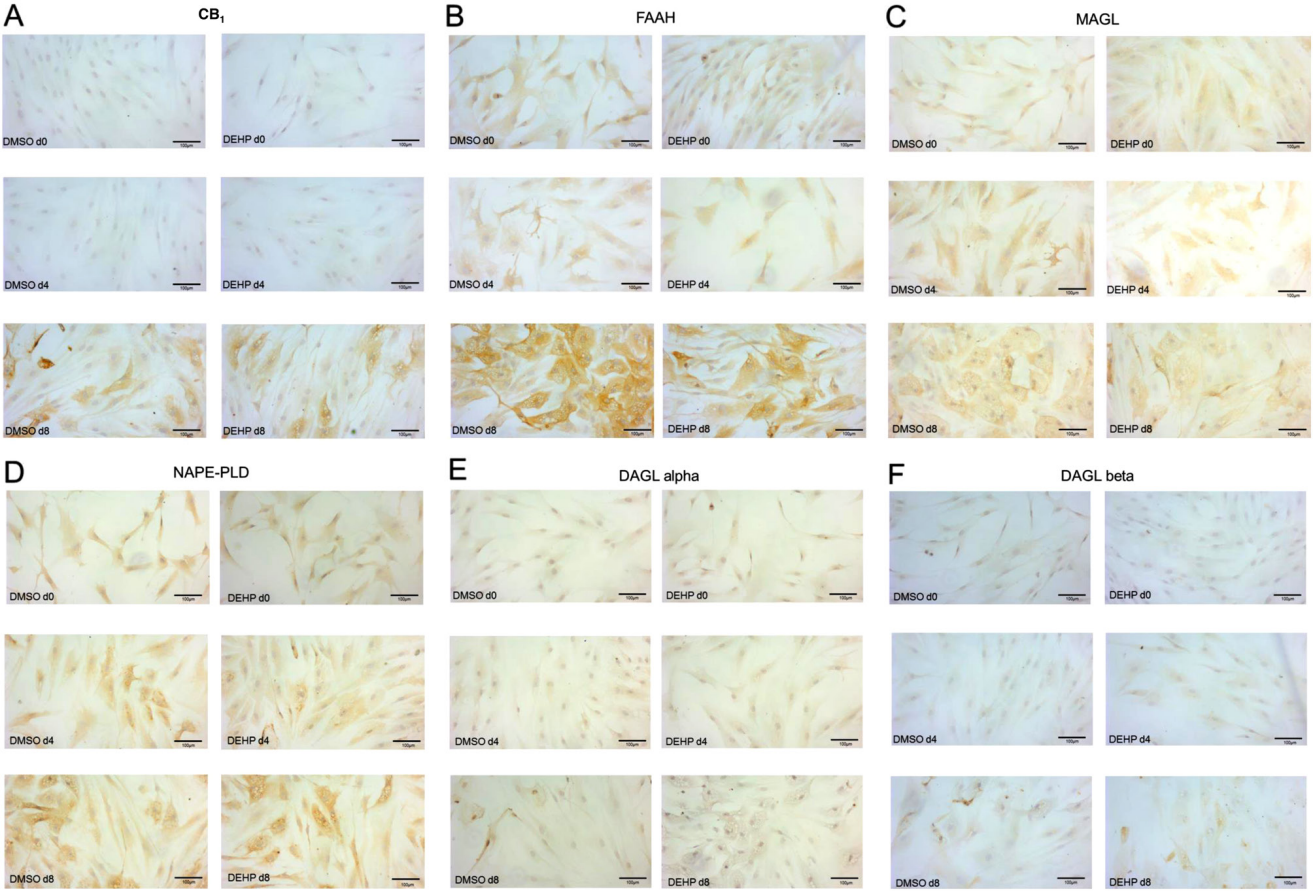
The localization of the ECS in the SGBS cell model with and without DEHP treatment. Immunohistochemical staining of CB_1_ (A), FAAH (B), MAGL (C), NAPE-PLD (D), DAGLalpha (E) and DAGLbeta (F) in SGBS cells at day 0, 4 and 8 after treatment with DMSO or DEHP (scale bar = 100 µm).

Furthermore, immunohistochemical studies were performed. During induction phase, SGBS cells grew mostly remaining spindle shaped. With increasing differentiation, cells became larger with expanded somata and visible lipid droplets. Additionally, only few isolated, very small cells were present in cultures (Fig. 3). All investigated ECS components were expressed in fully differentiated SGBS adipocytes. Comparing the data obtained from qRT-PCR, Western blot and immunohistochemistry describes an ECS component-specific characteristic pattern during adipogenesis with partial discrepancy. CB_1_ and FAAH immunoreactivities were evident in the somata of day 8-adipocytes with only very few positive cells at days 0 and 4 (Fig. 3A and B). A rather weak immunoreactivity was observed for MAGL at day 4. At day 8, MAGL was stained with stronger intensity mainly located around lipid droplets (Fig. 3C). NAPE-PLD immunoreactivity was found at day 4 and with much stronger intensity at day 8 (Fig. 3D). Whereas DAGLalpha positive cells were particularly abundant at days 0 and 4, a weak DAGLalpha immunoreactivity was observed at day 8 of adipogenesis (Fig. 3E). DAGLbeta-positive cells were more abundant and intensely stained than DAGLalpha positive cells (Fig. 3F). Notably, for all ECS components, no difference was detected between the DEHP-exposed and the DMSO-control group at all days investigated (Fig. 3A, B, C, D, E and F).

## Discussion

The present study aimed to investigate whether the obesogenic and endocrine-disrupting DEHP effects in adipocytes were mediated via the ECS. At first, we characterized the intrinsic ECS during normal adipogenic differentiation from SGBS preadipocytes to mature adipocytes. The receptors *CNR1* and *TRPV1* and the endocannabinoid-metabolizing enzymes *FAAH* and *MAGL* as well as the endocannabinoid-synthetizing enzymes *NAPE-PLD* and *DAGLalpha* were expressed. *CNR2* and *GPR55* – two additional receptors – were not detectable. The findings on *CNR2* are in agreement with data from bone marrow derived adipocytes (47). Also, expression and binding assays performed in human s.c. adipose tissue revealed a functional expression for CB_1_ and TRPV1, but not for CB_2_ (57). The presence of CB_2_ in adipose tissue has been a matter of controversy. An earlier study postulated contaminations with vascular, blood and immune cells as a potential source of positive CB_2_-findings (41). In our cell model, a contamination can be ruled out. By further analysing the expression pattern of *CNR1* and *TRPV1*, we found both receptors to be upregulated during the induction phase followed by a decrease during differentiation. For CB_1,_ an increasing immunoreactivity was detectable throughout maturation. In human bone marrow derived adipocytes, *CNR1* and *TRPV1* also increased after induction of adipogenesis. Additionally, a CB_1_-dependent inhibition of the differentiation was demonstrated, while otherwise AEA promoted adipogenesis by transactivation of PPARgamma (47). Comparing cannabinoid receptor expression in human primary fat cells before and during adipogenic differentiation, it was shown that *CNR1* was not expressed in preadipocytes, but rapidly appeared with differentiation, whereas *CNR2* started at low levels to become undetectable (41). In contrast, in preadipocytes and mature adipocytes isolated from human omental and s.c. adipose tissue, *CNR1* and *CNR2* were present in both cell types, but were more abundant in mature adipocytes. Interestingly, *CNR2* was much higher expressed than *CNR1* with functional proteins for both (58). A functional expression of CB_1_, CB_2_ and TRPV1 in murine 3T3-derived adipocytes was demonstrated during adipogenesis with increasing CB_1_ but declining CB_2_, whereas TRPV1 was unchanged (40). Investigating the protein expression pattern of the enzymes responsible for synthesis or degradation of main endocannabinoids namely 2-AG and AEA in the SGBS cell model showed: (a.) a slight increase in immunoreactivity of both DAGLalpha and MAGL for metabolizing 2-AG and (b.) no change in NAPE-PLD accompanied by an increase in FAAH immunoreaction for AEA during adipogenesis. The findings might point to a more or less constant 2-AG and a decrease in AEA levels. Studies with activity and expression data demonstrated a functional expression of enzymes involved in biosynthesis and hydrolysis of endocannabinoids in human s.c. and abdominal adipose tissue (41, 57). Differentiation of murine 3T3-derived adipocytes was accompanied by an increasing degradation of AEA by FAAH, whereas synthesis by NAPE-PLD was not influenced. Additionally, AEA was found to enhance – via CB_1_ – the insulin-regulated glucose uptake, that typical increases during adipogenic maturation (40). Furthermore, stimulation of murine 3T3-derived adipocytes with leptin decreased AEA and 2-AG levels (45). As adipogenic differentiation is paralleled by increasing leptin levels, the reduction of endocannabinoids by upregulation of endocannabinoid-metabolizing enzymes during adipogenesis may be the consequence of leptin-mediated regulation.

Concordant to previous data (50), the typical adipocyte markers leptin, adiponectin and *GLUT-* were upregulated during adipogenic maturation of SGBS adipocytes. Our investigations include not only the insulin-regulated glucose transporter *GLUT4* but also the basal glucose transporter *GLUT1*. In contrast to the increase of *GLUT4*, we found *GLUT1* to be reduced. This is in agreement with results of previous studies in human adipocyte precursor cells, assuming differentiation to be correlated with the development of insulin sensitivity in mature adipocytes (59, 60). As already described, DEHP exposure did not influence cell proliferation, but impaired adipogenic differentiation and lowered the lipid content of mature adipocytes (53). In proof of the efficacy of the present experiments, we investigated the DEHP action on adipokines. In agreement to our recent data (53), adiponectin was decreased and leptin was increased after DEHP exposure in mature SGBS adipocytes. Notably, in murine 3T3-derived adipocytes, DEHP reduced the cellular lipid content and adiponectin but increased the cell proliferation (14). In contrast, the higher number of adipocytes and the enhanced adipogenic differentiation of the murine mesenchymal stem cell line C3H/10T1/2 by DEHP exposure were associated with an increase of adiponectin expression (17). *In vivo*, DEHP exposure of mice and rats caused a gain of fat mass and a decrease of the serum adiponectin but increase of serum leptin (14, 15, 16, 61). Environmental contamination by DEHP is associated with human obesity (9, 12, 13). Interestingly, a positive correlation to the BMI and body fat was found for the circulating levels of endocannabinoids, whereas obesity was linked to a reduced adipose expression of *Cnr1* and *FAAH* (34, 35, 45). DEHP was found to interact with receptors of the ECS, namely CB_1_ and PPARs (48, 49). The herein investigated receptors and enzymes of the ECS were not altered by DEHP. As shown before, DEHP did not affect the protein amount of PPARalpha and PPARgamma in SGBS (53). However, DEHP-mediated alterations on PPARs was reported in both directions as assessed in rodent *in vitro* and *in vivo* experiments (14, 16, 17, 61), which points toward species-specific effects. Taken together, the absence of any effect on the expression of ECS enzymes by DEHP in the present study does not mean that DEHP has no impact on endocannabinoid metabolism. Further functional studies, for example, on enzyme activity will be needed to adequately address this aspect.

Summing up, in the human SGBS cell model an upregulation of the ECS receptors *CNR1* and *TRPV1* as well as the endocannabinoid-metabolizing enzymes *FAAH* and *MAGL* – presumably for reducing the endocannabinoid level in the differentiation process – was found during normal adipogenesis. As expected, the secretion of adiponectin and leptin was simultaneously increased. These data implicate the ECS to play a role in normal adipogenesis. As DEHP altered the level of adipokines secreted by mature adipocytes without affecting the intrinsic ECS, we conclude this DEHP-mediated endocrine impairment to be independent of the intrinsic ECS as endocrine modulator.

## Declaration of interest

The authors declare that there is no conflict of interest that could be perceived as prejudicing the impartiality of the research reported.

## Funding

Jana Ernst, Kristina Schädlich and Urszula Grabiec were supported by the Roux Programme of the Faculty of Medicine, Martin Luther University Halle-Wittenberg (JE FKZ 29/11, KS FKZ 26/06, UG FKZ 29/18). We acknowledge the financial support within the funding programme Open Access Publishing by the German Research Foundation (DFG).

## Author contribution statement

JE performed acquisition, analysis and interpretation of data, conception and design of the study, drafted the article and approved the final manuscript. UG performed acquisition, analysis and interpretation of data and approved the final manuscript. KF performed acquisition, analysis and interpretation of data and approved the final manuscript. FD was a project leader and performed revision and approved the final manuscript. KS was a project leader and performed conception and design of the study and revision and approved the final manuscript.
